# Supplementary material for: GAWMerge expands GWAS sample size and diversity by combining array-based genotyping and whole-genome sequencing
Source: Commun Biol. 2022 Aug 11;5:806. doi: 10.1038/s42003-022-03738-6 (PMC9372058; doi:10.1038/s42003-022-03738-6)
Supplement: Supplementary file 2 — Supplementary Information [file 42003_2022_3738_MOESM2_ESM.pdf]

Supplementary Figure 1: Utility of the empirical R^2 (ER^2) filter. Taking the test between COGEND and COPDGene EA sample comparison (Analysis 1 in the control of false positive tests) as an example, we conducted GWAS with COGEND EA array data as cases and COPDGene EA1 WGS data as controls. **(a)** Before applying the ER^2 filter, many false positives resulted due to the poor ER^2. **(b)** With the ER^2 filter of 0.9, the false positives were well controlled. **(c)** The histogram shows the distribution of ER^2 in all genotyped SNPs. **(d)** The scatter plot of the outlier SNPs with $ER^2 < 0.9$ were evenly distributed across the genome. The Y-axis is the value of ER^2.

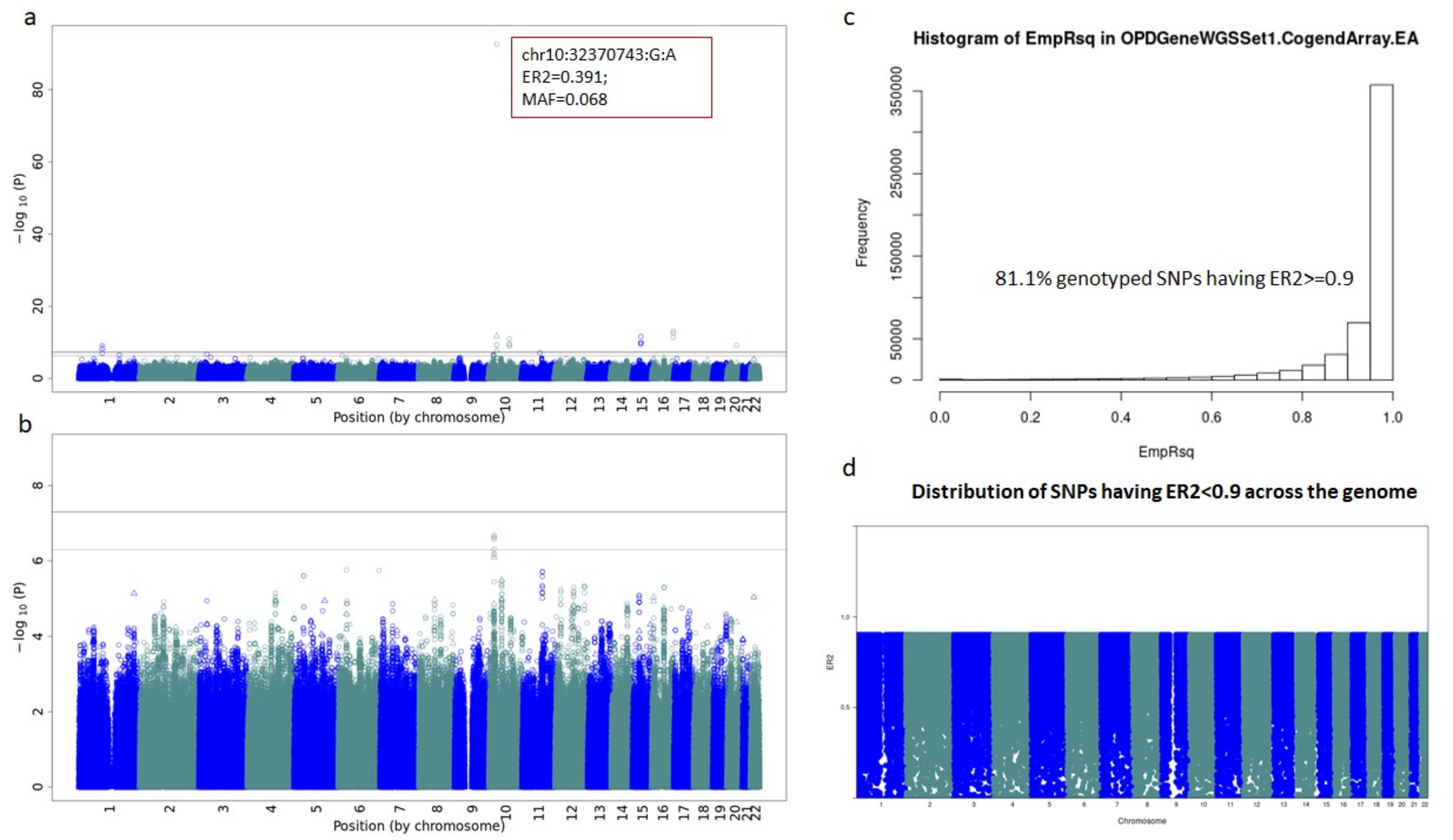
a
chr10:32370743:G:A
ER2=0.391;
MAF=0.068
−log 10 (P)
Position (by chromosome)
b
c
Histogram of EmpRsq in OPDGeneWGSSet1.CogendArray.EA
Frequency
81.1% genotyped SNPs having ER2>=0.9
EmpRsq
d
Distribution of SNPs having ER2<0.9 across the genome
ER2
Chromosome

Supplementary Figure 2: Utility of R^2 Difference Filter. Taking the test between COGEND and COPDGene EA sample comparison (Analysis 1 in the control of false positive tests) as an example, we conducted GWAS with COGEND EA array data as cases and COPDGene EA1 WGS data as controls. **(a)** The QQ plot shows the P-values for the subset of SNPs with $|R^2_{array} - R^2_{WGS}| \geq 0.1$ has problematic inflation (black dots), but good for other SNPs (orange and blue dots). **(b)** The histogram shows the distribution of Rsq_diff ($|R^2_{array} - R^2_{WGS}|$) in all SNPs. **(c)** The scatter plot of the outlier SNPs with $|R^2_{array} - R^2_{WGS}| \geq 0.1$ are evenly distributed across the genome. The Y-axis is the value of Rsq_diff.

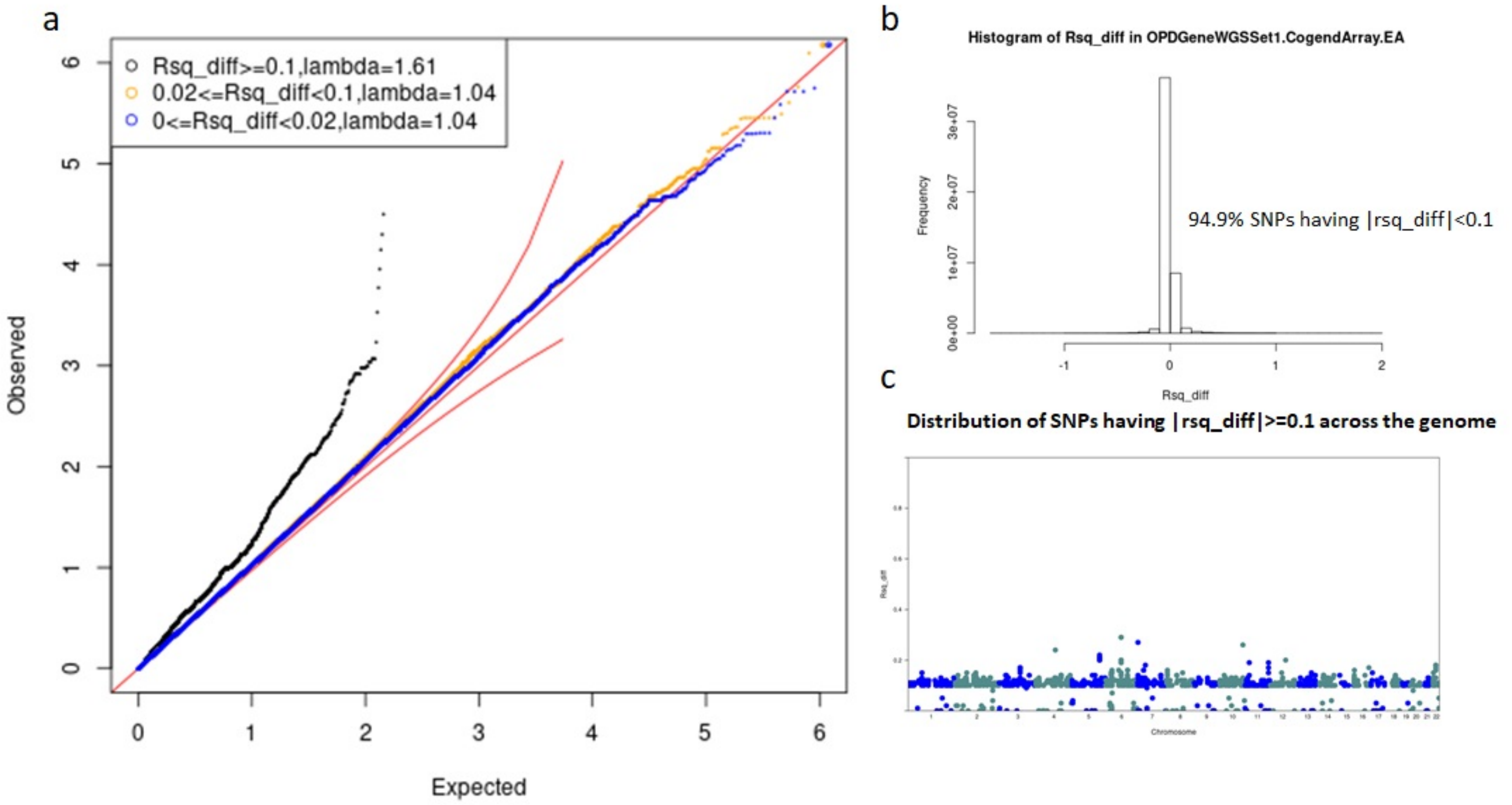
a
Rsq_diff>=0.1,lambda=1.61
0.02<=Rsq_diff<0.1,lambda=1.04
0<=Rsq_diff<0.02,lambda=1.04
Observed
Expected
0
1
2
3
4
5
6
b
Histogram of Rsq_diff in OPDGeneWGSSet1.CogendArray.EA
Frequency
0e+00
1e+07
2e+07
3e+07
94.9% SNPs having |rsq_diff|<0.1
-1
0
1
2
Rsq_diff
c
Distribution of SNPs having |rsq_diff|>=0.1 across the genome
Chromosome

Supplementary Figure 3: False Positives with TOPMed phased data. Manhattan plot comparing WGS phased vs array data from the same set of COPDGene samples. The WGS data is phased by TOPMed based on all genotypes on all samples within the program. Therefore, the array and WGS data have been phased with different single nucleotide polymorphisms and sample set. We believe the difference in phasing is the cause of the false positives and these are corrected in the results shown in Supplementary Figure 4.

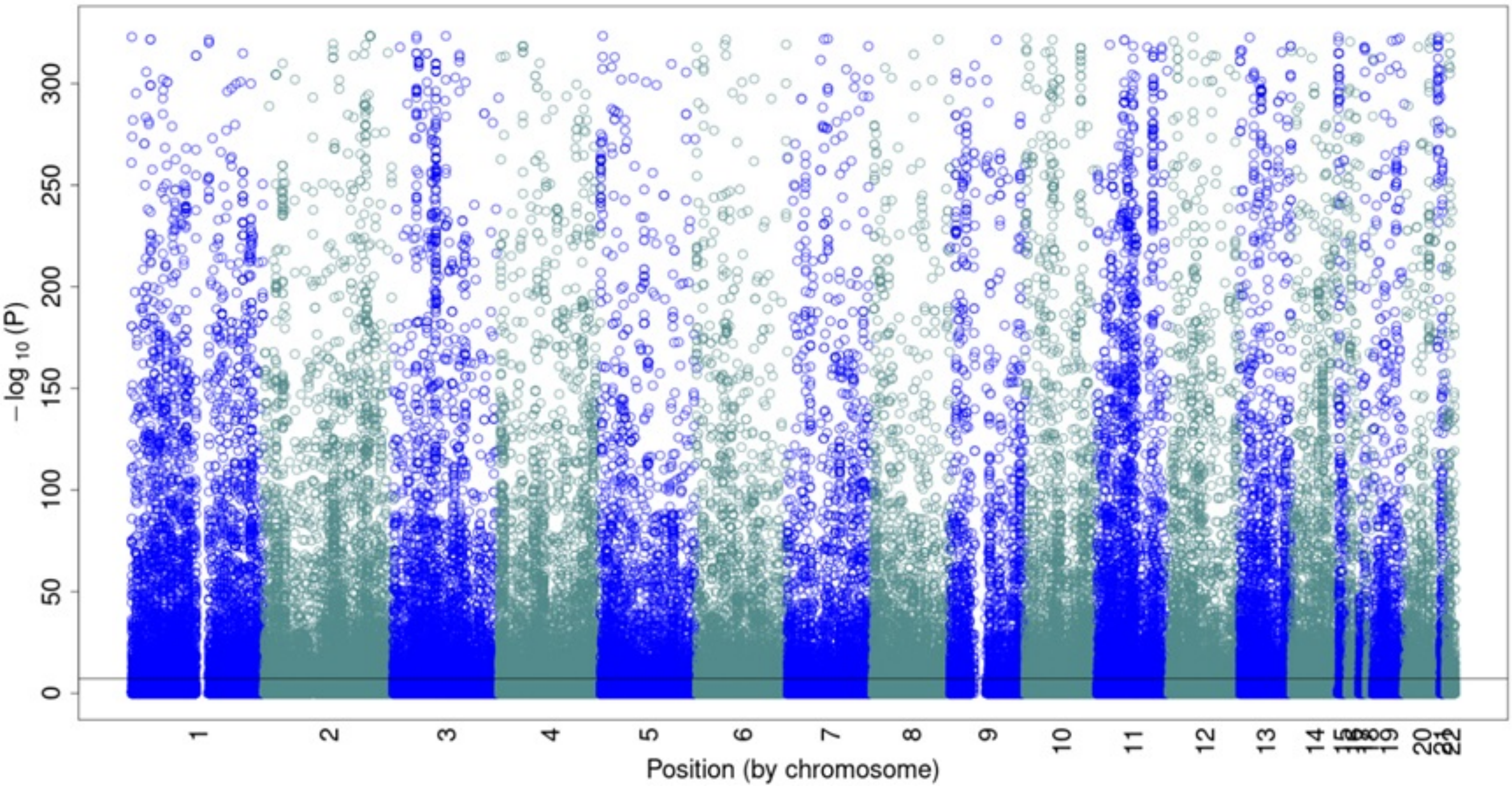

−log 10 (P)
0
50
100
150
200
250
300
Position (by chromosome)
1
2
3
4
5
6
7
8
9
10
11
12
13
14
15
19
20
22

Supplementary Figure 4: Technical reproducibility across genotyping technology. Manhattan plots comparing WGS vs. array genotyping data from the same set of COPDGene samples of **(a)** European ancestry (N=6,501) and **(b)** African American ancestries (N=3,235) after applying GAWMerge.

a

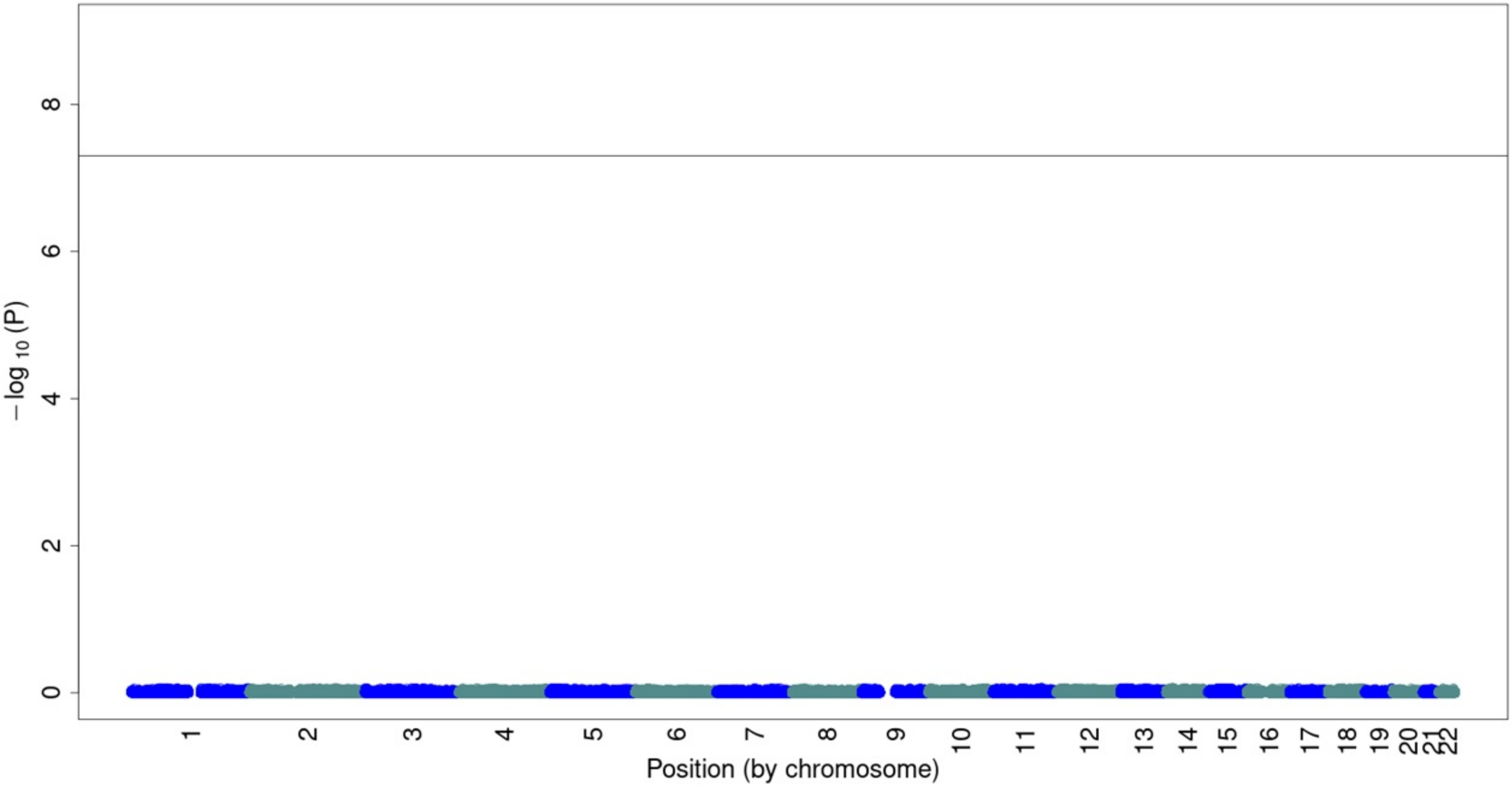

8
6
4
2
0
−log 10 (P)
1
2
3
4
5
6
7
8
9
10
11
12
13
14
15
16
17
18
19
20
21
22
Position (by chromosome)

b

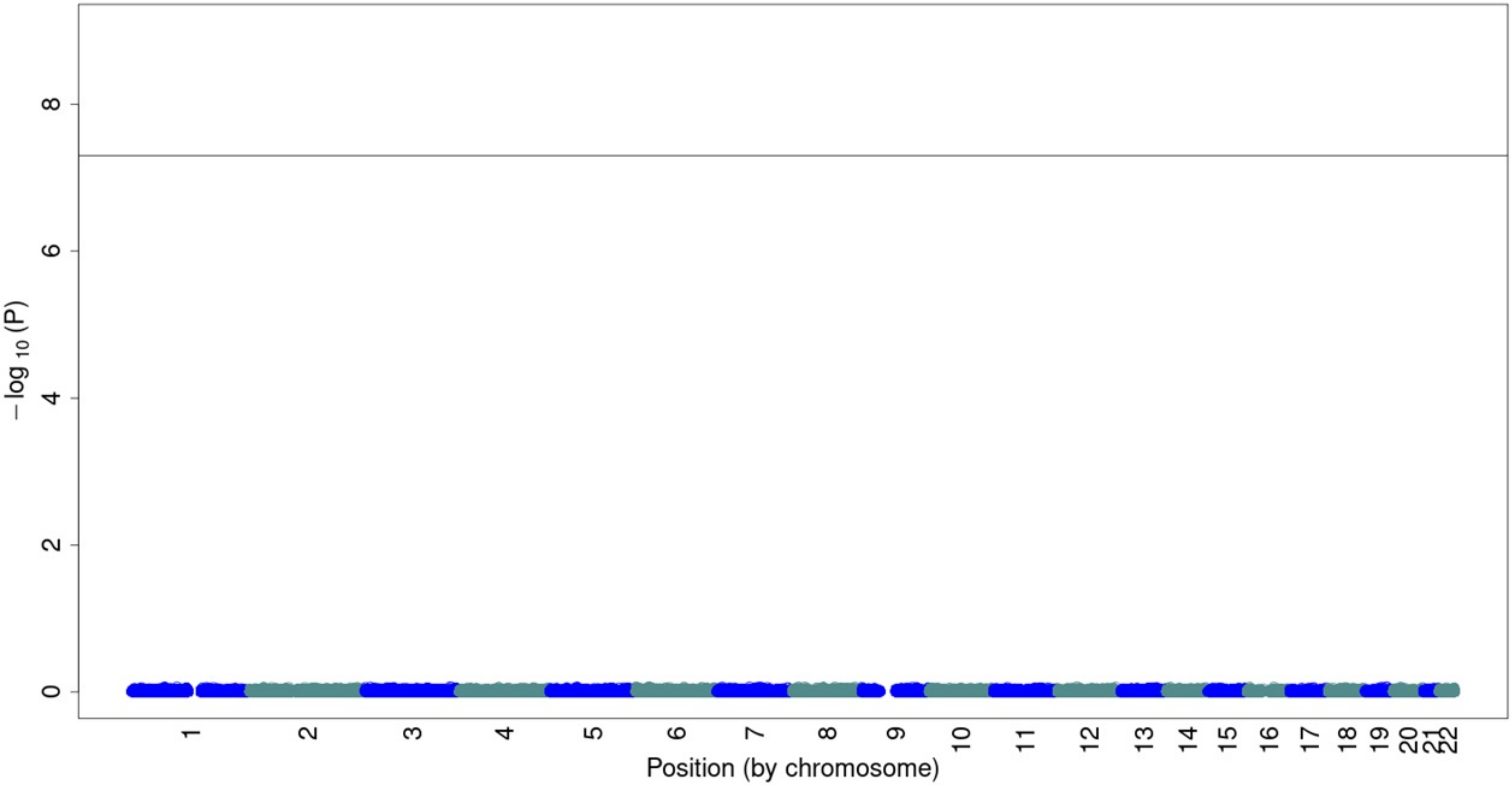

8
6
4
2
0
−log 10 (P)
1
2
3
4
5
6
7
8
9
10
11
12
13
14
15
16
17
18
19
20
21
22
Position (by chromosome)

Supplementary Figure 5: Controlling type 1 error across genotype technology. Manhattan and quantile-quantile (QQ) plots for the analyses to control type I error for the **(a)** COGEND EA array data (N=1,961) vs. COPDGene EA1 WGS data (N=3,251), **(b)** COPDGene EA2 array data (N=3,251) vs. ECLIPSE EA WGS data (N=1,461), and **(c)** COGEND AA array data (N=712) Vs. COPDGene AA WGS data (N=1,710) analyses. As all of the cohorts, COGEND, COPDGene, and ECLIPSE are smoking cohorts along with the COPD case distributed across both classes being tested, we see no genome-wide signal, thus controlling type 1 error.

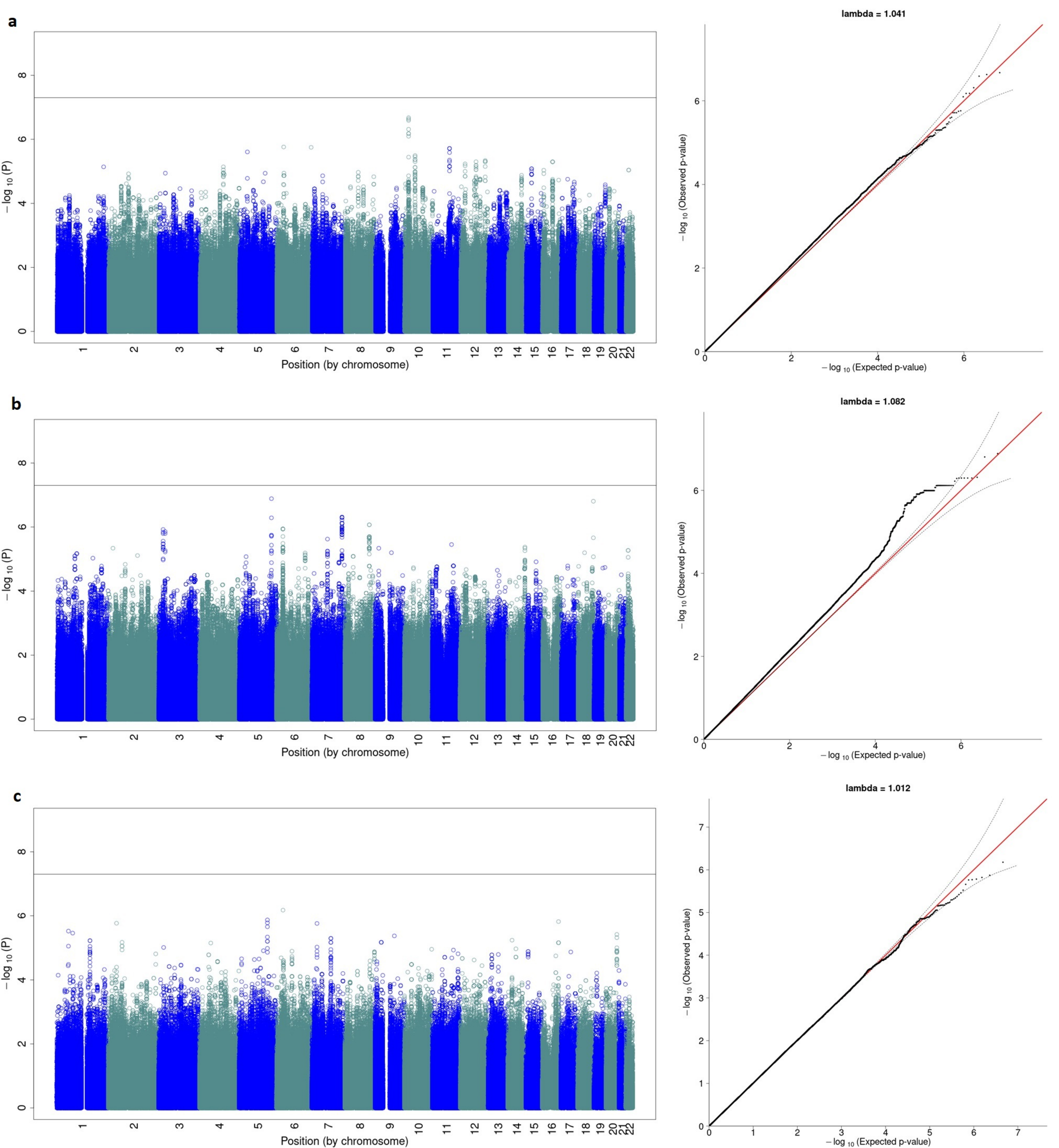
a
b
c
−log 10 (P)
Position (by chromosome)
lambda = 1.041
lambda = 1.082
lambda = 1.012
−log 10 (Observed p-value)
−log 10 (Expected p-value)

Supplementary Figure 6: Recovery of known loci across technology. Manhattan and quantile-quantile (QQ) plots for the analyses to recover COPD GWAS signals for the **(a)** COGEND EA array data as smoking controls (N=1,961) and COPDGene EA with COPD WGS data as cases (N=2,736), **(b)** ECLIPSE EA with COPD array data as cases (N=1,764) and COPDGene EA with no COPD WGS data as smoking controls (N=2,475), and **(c)** COGEND AA array data as smoking controls (N=712) and COPDGene AA with COPD WGS data as cases (N=813) analyses.

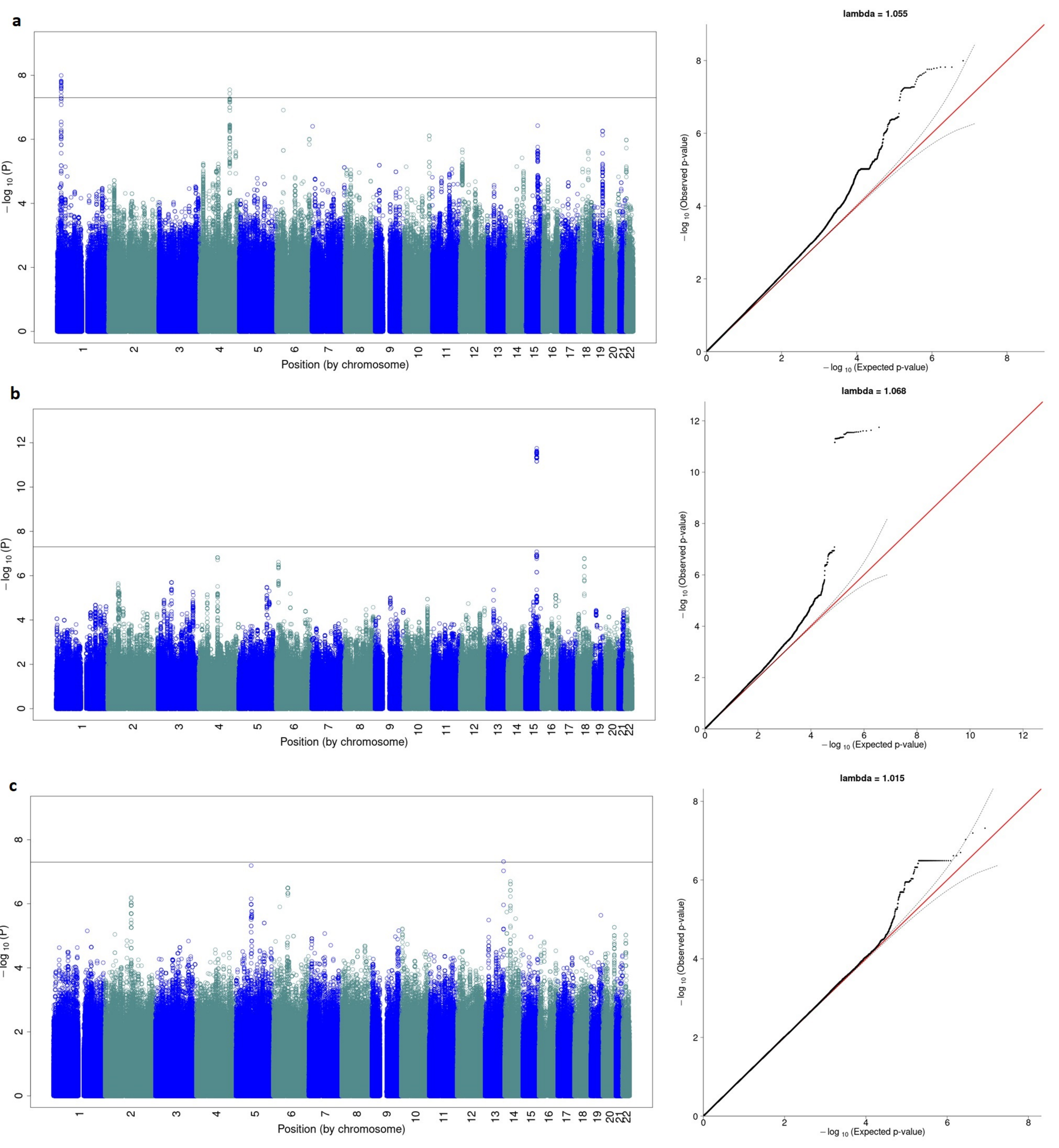
a
lambda = 1.055
b
lambda = 1.068
c
lambda = 1.015
− log 10 (P)
Position (by chromosome)
− log 10 (Observed p-value)
− log 10 (Expected p-value)

Supplementary Tables

Supplementary Table 1: Overlap of TOPMed WGS data with the targeted arrays.

Array type	#SNPs on the array	#SNPs overlapped with WGS data	Overlap%
MetaboChip array	196,725	188,086	95.61%
Immunochip array	253,702	246,668	97.23%
OncoArray	499,170	484,288	97.02%

Supplementary Table 2: All genome-wide significant ($P < 5 \times 10^{-8}$) peaks from the recovery of GWAS signals for COPD meta-analysis.

MarkerName	Allele1	Freq1	OR	P-value	Direction
15:78817929:C:T	t	0.61	0.78	1.33E-16	---
15:78857986:C:G	c	0.64	0.78	1.60E-16	---
15:78867482:A:C	a	0.63	0.78	1.91E-16	---
15:78898723:C:T	t	0.40	1.28	3.35E-16	+++
15:78802869:C:T	t	0.62	0.78	1.05E-15	---
15:78900647:A:G	a	0.65	0.78	1.48E-15	---
15:78901113:C:T	t	0.35	1.28	1.48E-15	+++
15:78900701:T:G	t	0.65	0.78	1.48E-15	---
15:78900650:C:T	t	0.35	1.28	1.48E-15	+++
15:78816057:G:T	t	0.36	1.28	1.67E-15	+++
15:78899560:CAA:C	caa	0.64	0.78	1.67E-15	---
15:78899003:C:A	a	0.35	1.28	1.77E-15	+++
15:78898932:C:G	c	0.65	0.78	2.14E-15	---
15:78859605:AAAAAG:A	a	0.36	1.28	2.20E-15	+++
15:78850501:AT:A	a	0.36	1.28	2.94E-15	+++
15:78862064:C:T	t	0.36	1.29	3.16E-15	+++
15:78900908:C:T	t	0.35	1.28	3.58E-15	+++
15:78868636:G:A	a	0.36	1.29	3.77E-15	+++
15:78911181:T:C	t	0.58	0.79	4.38E-15	---
15:78865197:A:G	a	0.65	0.78	6.00E-15	---
15:78886198:C:T	t	0.36	1.29	6.03E-15	+++
15:78882925:G:A	a	0.36	1.29	6.39E-15	+++
15:78915370:CT:C	ct	0.58	0.79	6.75E-15	---
15:78866445:G:A	a	0.35	1.28	7.05E-15	+++
15:78906177:A:T	a	0.64	0.78	7.39E-15	---
15:78806023:T:C	t	0.64	0.78	7.43E-15	---
15:78862453:C:A	a	0.36	1.28	7.43E-15	+++
15:78886947:G:A	a	0.36	1.29	7.45E-15	+++
15:78813155:G:A	a	0.36	1.28	8.64E-15	+++
15:78873993:A:T	a	0.64	0.78	9.22E-15	---
15:78849034:T:C	t	0.64	0.79	9.24E-15	---
15:78851615:G:A	a	0.36	1.27	1.30E-14	+++
15:78857939:T:G	t	0.64	0.78	1.66E-14	---
15:78801394:A:C	a	0.65	0.79	1.82E-14	---
15:78912710:T:TGCGCGGGGCAGGGCGACGGGCA	t	0.64	0.78	1.91E-14	---
15:78878541:G:A	a	0.36	1.27	2.01E-14	+++
15:78877381:C:A	a	0.36	1.27	2.20E-14	+++
15:78894339:G:A	a	0.35	1.27	2.50E-14	+++
15:78911672:G:C	c	0.36	1.27	2.78E-14	+++

15:78814046:G:A	a	0.63	0.79	3.72E-14	---
15:78828086:G:T	t	0.36	1.26	8.35E-14	+++
15:78849779:C:T	t	0.36	1.26	1.34E-13	+++
15:78896129:A:G	a	0.64	0.80	2.89E-13	---
15:78826180:G:A	a	0.64	0.80	1.10E-12	--+
15:78915872:C:A	a	0.38	1.24	3.43E-12	+++
15:78915864:G:A	a	0.44	1.23	5.41E-12	+++
15:78914534:C:T	t	0.38	1.23	1.32E-11	+++
4:89866713:T:C	t	0.57	0.83	2.66E-10	---
4:89860830:C:T	t	0.43	1.21	2.86E-10	+++
4:89750361:A:G	a	0.66	0.81	4.13E-10	--?
4:89869332:T:C	t	0.55	0.83	4.19E-10	--+
4:89870964:C:T	t	0.45	1.21	4.70E-10	++-
4:89869078:C:T	t	0.44	1.20	6.10E-10	++-
4:89873092:A:G	a	0.56	0.83	8.89E-10	--+
4:89869918:G:A	a	0.44	1.20	8.89E-10	++-
4:89872176:G:A	a	0.44	1.20	1.15E-09	++-
4:145463567:C:T	t	0.38	0.83	2.12E-09	---
4:145454374:C:T	t	0.37	0.83	2.72E-09	---
4:145449525:G:GAT	g	0.63	1.20	3.51E-09	+++
4:145437014:T:C	t	0.43	1.19	3.70E-09	+++
4:145465768:T:C	t	0.63	1.20	3.73E-09	+++
4:145462588:A:C	a	0.63	1.20	3.73E-09	+++
4:145458484:T:C	t	0.63	1.20	3.73E-09	+++
4:145460230:A:G	a	0.63	1.20	3.73E-09	+++
4:145455550:C:T	t	0.37	0.83	3.73E-09	---
4:145464885:A:G	a	0.59	1.19	3.98E-09	+++
4:145471245:A:T	a	0.63	1.20	3.98E-09	+++
4:145463521:G:A	a	0.37	0.83	4.85E-09	---
4:145463533:T:C	t	0.63	1.20	4.85E-09	+++
4:145460338:A:G	a	0.63	1.20	4.85E-09	+++
4:145465461:C:G	c	0.63	1.20	4.85E-09	+++
4:145434901:A:G	a	0.62	1.20	4.98E-09	+++
4:145454232:C:T	t	0.37	0.84	5.07E-09	---
4:145454964:T:C	t	0.63	1.20	6.70E-09	+++
4:145465491:T:G	t	0.59	1.19	6.80E-09	+++
4:145445779:A:T	a	0.61	1.21	6.88E-09	++?
4:145471255:CA:C	ca	0.63	1.20	7.42E-09	+++
4:145469968:G:T	t	0.37	0.84	7.79E-09	---
4:145474297:G:A	a	0.37	0.84	7.79E-09	---
4:145478662:C:T	t	0.37	0.84	8.32E-09	---
4:145473196:G:A	a	0.38	0.84	1.10E-08	---
4:89885714:T:C	t	0.48	0.84	1.17E-08	---

4:145436324:T:C	t	0.63	1.19	1.27E-08	+++
4:145436894:G:C	c	0.37	0.84	1.27E-08	---
15:78910258:C:T	t	0.21	0.81	1.31E-08	---
15:78910598:C:CT	ct	0.22	0.82	1.46E-08	---
4:145434744:A:G	a	0.62	1.19	1.63E-08	+++
15:78799060:ACT:A	a	0.60	1.19	1.80E-08	+++
19:41302706:C:T	t	0.54	1.18	1.91E-08	+++
4:145470657:T:A	a	0.37	0.84	1.96E-08	---
4:145478201:C:T	t	0.37	0.84	1.96E-08	---
4:145479880:T:C	t	0.63	1.19	1.96E-08	+++
4:145478049:C:G	c	0.63	1.19	1.96E-08	+++
4:145452783:C:T	t	0.57	0.85	2.19E-08	---
15:78899719:G:A	a	0.77	1.22	2.25E-08	+++
4:89884114:TG:T	t	0.52	1.18	2.25E-08	+++
4:89883979:C:T	t	0.52	1.18	2.25E-08	+++
15:78896547:G:A	a	0.78	1.22	2.33E-08	+++
15:78899213:T:C	t	0.22	0.82	2.36E-08	---
15:78912943:G:A	a	0.21	0.82	2.49E-08	---
15:78903987:T:C	t	0.22	0.82	2.59E-08	---
15:78888234:A:ACCCC	a	0.78	1.22	2.75E-08	+++
4:145463231:G:A	a	0.38	0.85	2.75E-08	---
4:145467212:A:T	a	0.62	1.18	2.87E-08	+++
15:78796104:C:T	t	0.59	1.19	2.88E-08	+++
15:78754000:A:G	a	0.41	0.84	2.88E-08	---
4:89924725:A:C	a	0.55	1.19	3.06E-08	++?
15:78765290:T:G	t	0.41	0.84	3.08E-08	---
4:89885086:G:T	t	0.51	1.18	3.11E-08	+++
4:145445117:A:G	a	0.42	1.18	3.12E-08	+++
15:78751961:C:T	t	0.59	1.19	3.18E-08	+++
15:78779510:T:A	a	0.59	1.19	3.31E-08	+++
15:78789223:G:A	a	0.59	1.19	3.31E-08	+++
4:89908381:GA:G	g	0.33	0.84	3.31E-08	---
15:78793921:C:T	t	0.59	1.19	3.33E-08	+++
15:78792398:T:C	t	0.41	0.84	3.33E-08	---
15:78747916:AAAAAAG:A	a	0.59	1.19	3.35E-08	+++
4:145479139:A:G	a	0.59	1.18	3.37E-08	+++
4:145480780:A:G	a	0.59	1.18	3.37E-08	+++
15:78845110:A:G	a	0.79	1.22	3.51E-08	+++
15:78837673:C:T	t	0.21	0.82	3.58E-08	---
15:78782095:C:T	t	0.59	1.19	3.64E-08	+++
15:78789488:C:T	t	0.59	1.19	3.64E-08	+++
4:145468791:G:A	a	0.57	0.85	3.66E-08	---
15:78767346:G:A	a	0.59	1.19	3.67E-08	+++

4:145445694:T:A	a	0.57	0.85	3.75E-08	---
4:145444039:A:C	a	0.43	1.18	3.75E-08	+++
4:145464074:T:A	a	0.40	0.85	4.00E-08	---
4:145462364:G:A	a	0.40	0.85	4.00E-08	---
4:89765661:G:C	c	0.35	1.18	4.06E-08	++-
15:78908565:C:T	t	0.21	0.80	4.18E-08	--?
15:78769130:A:G	a	0.41	0.84	4.30E-08	---
4:89924434:GAA:G	g	0.46	0.85	4.43E-08	---
4:145425936:A:G	a	0.64	1.19	4.57E-08	++-
15:78766194:T:A	a	0.59	1.18	4.73E-08	+++
15:78767850:C:T	t	0.59	1.18	4.73E-08	+++
15:78868398:C:T	t	0.25	0.83	4.79E-08	---

Supplementary Table 3: Number of samples and SNPs in each step of the analysis. Taking the test between COPDGene EA array data and WGS data (Analysis 1 in the technical comparison) as an example, the number of samples and SNPs are recorded for each step. Throughout the table, the steps are as labeled in **Figure 1**.

	**COPDGene EA array data**		**COPDGene EA WGS data**	
	**#Samples**	**#SNPs**	**#Samples**	**#SNPs**
Step 1: Original array and WGS data	6,670	648,530	6,507	626,638,014
Step 2: After Extraction of array SNPs from WGS	6,670	648,530	6,507	630,947
Step 3: After QC	6,664	639,505	6,507	614,283
	**Merged #Samples**		**Merged #SNPs**	
Step 5: After merging, input for 1st imputation	6,501		557,746	
Step 6: Input for 2nd imputation	6,501		484,502	
Step 7: After 2nd imputation	6,501		47,109,431	
Step 8: After all filtering	6,501		6,974,901	

Complete List of NHLBI Trans-Omics for Precision Medicine (TOPMed) Consortium Members

Namiko Abe[10], Gonçalo Abecasis[11], Francois Aguet[12], Christine Albert[13], Laura Almasy[14], Alvaro Alonso[15], Seth Ament[16], Peter Anderson[17], Pramod Anugu[18], Deborah Applebaum-Bowden[19], Kristin Ardlie[12], Dan Arking[20], Donna K Arnett[21], Allison Ashley-Koch[22], Stella Aslibekyan[23], Tim Assimes[24], Paul Auer[25], Dimitrios Avramopoulos[20], Najib Ayas[26], Adithya Balasubramanian[27], John Barnard[28], Kathleen Barnes[29], R. Graham Barr[30], Emily Barron-Casella[20], Lucas Barwick[31], Terri Beaty[20], Gerald Beck[32], Diane Becker[33], Lewis Becker[20], Rebecca Beer[34], Amber Beitelshees[16], Emelia Benjamin[35], Takis Benos[36], Marcos Bezerra[37], Larry Bielak[11], Joshua Bis[38], Thomas Blackwell[11], John Blangero[39], Eric Boerwinkle[40], Donald W. Bowden[41], Russell Bowler[42], Jennifer Brody[17], Ulrich Broeckel[43], Jai Broome[17], Deborah Brown[44], Karen Bunting[10], Esteban Burchard[45], Carlos Bustamante[46], Erin Buth[47], Brian Cade[48], Jonathan Cardwell[49], Vincent Carey[50], Julie Carrier[51], April Carson[52], Cara Carty[53], Richard Casaburi[54], Juan P Casas Romero[50], James Casella[20], Peter Castaldi[55], Mark Chaffin[12], Christy Chang[16], Yi-Cheng Chang[56], Daniel Chasman[57], Sameer Chavan[49], Bo-Juen Chen[10], Wei-Min Chen[58], Yii-Der Ida Chen[59], Seung Hoan Choi[12], Lee-Ming Chuang[60], Mina Chung[61], Ren-Hua Chung[62], Clary Clish[63], Suzy Comhair[64], Matthew Conomos[47], Elaine Cornell[65], Adolfo Correa[66], Carolyn Crandall[54], James Crapo[67], L. Adrienne Cupples[68], Joanne Curran[69], Jeffrey Curtis[70], Brian Custer[71], Coleen Damcott[16], Dawood Darbar[72], Sean David[73], Colleen Davis[17], Michelle Daya[49], Mariza de Andrade[74], Lisa de las Fuentes[75], Paul de Vries[76], Michael DeBaun[77], Ranjan Deka[78], Dawn DeMeo[50], Scott Devine[16], Huyen Dinh[27], Harsha Doddapaneni[27], Qing Duan[79], Shannon Dugan-Perez[27], Ravi Duggirala[80], Jon Peter Durda[65], Susan K. Dutcher[81], Charles Eaton[82], Lynette Ekunwe[18], Adel El Boueiz[83], Patrick Ellinor[84], Leslie Emery[17], Serpil Erzurum[28], Charles Farber[58], Jesse Farek[27], Tasha Fingerlin[85], Matthew Flickinger[11], Myriam Fornage[40], Nora Franceschini[86], Chris Frazar[17], Mao Fu[16], Stephanie M. Fullerton[17], Lucinda Fulton[87], Stacey Gabriel[12], Weiniu Gan[34], Shanshan Gao[49], Yan Gao[18], Margery Gass[88], Heather Geiger[89], Bruce Gelb[90], Mark Geraci[36], Soren Germer[10], Robert Gerszten[91], Auyon Ghosh[50], Richard Gibbs[27], Chris Gignoux[24], Mark Gladwin[36], David Glahn[92], Stephanie Gogarten[17], Da-Wei Gong[16], Harald Goring[93], Sharon Graw[94], Kathryn J. Gray[95], Daniel Grine[49], Colin Gross[11], C. Charles Gu[87], Yue Guan[16], Xiuqing Guo[59], Namrata Gupta[12], David M. Haas[96], Jeff Haessler[88], Michael Hall[97], Yi Han[27], Patrick Hanly[98], Daniel Harris[99], Nicola L. Hawley[100], Jiang He[101], Ben Heavner[47], Susan Heckbert[102], Ryan Hernandez[45], David Herrington[103], Craig Hersh[104], Bertha Hidalgo[23], James Hixson[40], Brian Hobbs[50], Elliott Hong[16], Karin Hoth[105], Chao (Agnes) Hsiung[106], Jianhong Hu[27], Yi-Jen Hung[107], Haley Huston[108], Chii Min Hwu[109], Marguerite Ryan Irvin[23], Rebecca Jackson[110], Deepti Jain[17], Cashell Jaquish[111], Jill Johnsen[112], Andrew Johnson[34], Craig Johnson[17], Rich Johnston[15], Kimberly Jones[20], Hyun Min Kang[113], Robert Kaplan[114], Sharon Kardia[11], Shannon Kelly[45], Eimear Kenny[90], Michael Kessler[16], Alyna Khan[17], Ziad Khan[27], Wonji Kim[115], John Kimoff[116], Greg Kinney[117], Barbara Konkle[118], Charles Kooperberg[88], Holly Kramer[119], Christoph Lange[120], Ethan Lange[49], Leslie Lange[121], Cathy Laurie[17], Cecelia Laurie[17], Meryl LeBoff[50], Jiwon Lee[50], Sandra Lee[27], Wen-Jane Lee[109], Jonathon LeFaive[11], David Levine[17], Dan Levy[34], Joshua Lewis[16], Xiaohui Li[59], Yun Li[79], Henry Lin[59], Honghuang Lin[122], Xihong Lin[123], Simin Liu[124], Yongmei Liu[125], Yu Liu[126], Ruth J.F. Loos[127], Steven Lubitz[84], Kathryn Lunetta[128], James Luo[34], Ulysses Magalang[129], Michael Mahaney[69], Barry Make[20], Ani Manichaikul[58], Alisa Manning[130], JoAnn Manson[50], Lisa Martin[131], Melissa Marton[89], Susan Mathai[49], Rasika Mathias[20], Susanne May[47], Patrick McArdle[16], Merry-Lynn McDonald[132], Sean McFarland[115], Stephen McGarvey[133], Daniel McGoldrick[134], Caitlin McHugh[47], Becky McNeil[135], Hao Mei[18], James Meigs[136], Vipin Menon[27], Luisa Mestroni[94], Ginger Metcalf[27], Deborah A Meyers[137], Emmanuel Mignot[138], Julie Mikulla[34], Nancy Min[18], Mollie Minear[139], Ryan L Minster[36], Braxton D. Mitchell[16], Matt Moll[55], Zeineen Momin[27], May E. Montasser[16], Courtney Montgomery[140], Donna Muzny[27], Josyf C Mychaleckyj[58], Girish Nadkarni[90],

Rakhi Naik[20], Take Naseri[141], Pradeep Natarajan[12], Sergei Nekhai[142], Sarah C. Nelson[47], Bonnie Neltner[49], Caitlin Nessner[27], Deborah Nickerson[143], Osuji Nkechinyere[27], Kari North[79], Jeff O'Connell[144], Tim O'Connor[16], Heather Ochs-Balcom[145], Geoffrey Okwuonu[27], Allan Pack[146], David T. Paik[147], Nicholette Palmer[148], James Pankow[149], George Papanicolaou[34], Cora Parker[150], Gina Peloso[151], Juan Manuel Peralta[80], Marco Perez[24], James Perry[16], Ulrike Peters[152], Patricia Peyser[11], Lawrence S Phillips[15], Jacob Pleiness[11], Toni Pollin[16], Wendy Post[153], Julia Powers Becker[154], Meher Preethi Boorgula[49], Michael Preuss[90], Bruce Psaty[17], Pankaj Qasba[34], Dandi Qiao[50], Zhaohui Qin[15], Nicholas Rafaels[155], Laura Raffield[156], Mahitha Rajendran[27], Vasan S. Ramachandran[128], D.C. Rao[87], Laura Rasmussen-Torvik[157], Aakrosh Ratan[58], Susan Redline[55], Robert Reed[16], Catherine Reeves[158], Elizabeth Regan[67], Alex Reiner[159], Muagututi'a Sefuiva Reupena[160], Ken Rice[17], Stephen Rich[58], Rebecca Robillard[161], Nicolas Robine[89], Dan Roden[162], Carolina Roselli[12], Jerome Rotter[163], Ingo Ruczinski[20], Alexi Runnels[89], Pamela Russell[49], Sarah Ruuska[108], Kathleen Ryan[16], Ester Cerdeira Sabino[164], Danish Saleheen[30], Shabnam Salimi[165], Sejal Salvi[27], Steven Salzberg[20], Kevin Sandow[166], Vijay G. Sankaran[167], Jireh Santibanez[27], Karen Schwander[87], David Schwartz[49], Frank Sciurba[36], Christine Seidman[168], Jonathan Seidman[169], Frédéric Sériès[170], Vivien Sheehan[171], Stephanie L. Sherman[172], Amol Shetty[16], Aniket Shetty[49], Wayne Hui-Heng Sheu[109], M. Benjamin Shoemaker[173], Brian Silver[174], Robert Skomro[175], Jennifer Smith[11], Josh Smith[17], Nicholas Smith[102], Tanja Smith[10], Sylvia Smoller[114], Beverly Snively[176], Michael Snyder[24], Tamar Sofer[50], Nona Sotoodehnia[17], Adrienne M. Stilp[17], Garrett Storm[177], Elizabeth Streeten[16], Jessica Lasky Su[178], Yun Ju Sung[87], Jody Sylvia[50], Adam Szpiro[17], Daniel Taliun[11], Hua Tang[179], Margaret Taub[20], Kent D. Taylor[180], Matthew Taylor[94], Simeon Taylor[16], Marilyn Telen[22], Timothy A. Thornton[17], Machiko Threlkeld[181], Lesley Tinker[182], David Tirschwell[17], Sarah Tishkoff[183], Hemant Tiwari[184], Catherine Tong[185], Russell Tracy[186], Michael Tsai[149], Dhananjay Vaidya[20], David Van Den Berg[187], Peter VandeHaar[11], Scott Vrieze[149], Tarik Walker[49], Robert Wallace[105], Avram Walts[49], Fei Fei Wang[17], Heming Wang[188], Jiongming Wang[11], Karol Watson[54], Jennifer Watt[27], Daniel E. Weeks[36], Joshua Weinstock[113], Bruce Weir[17], Scott T Weiss[189], Lu-Chen Weng[84], Jennifer Wessel[190], Cristen Willer[70], Kayleen Williams[47], L. Keoki Williams[191], Carla Wilson[50], James Wilson[192], Lara Winterkorn[89], Quenna Wong[17], Joseph Wu[147], Huichun Xu[16], Lisa Yanek[20], Ivana Yang[49], Ketian Yu[11], Seyedeh Maryam Zekavat[12], Yingze Zhang[193], Snow Xueyan Zhao[67], Wei Zhao[194], Xiaofeng Zhu[195], Michael Zody[10], Sebastian Zoellner[11]

[10]New York Genome Center, New York, NY, USA.[11]University of Michigan, Ann Arbor, MI, USA.[12]Broad Institute, Cambridge, MA, USA.[13]Cedars Sinai, Boston, MA, USA.[14]Children's Hospital of Philadelphia, University of Pennsylvania, Philadelphia, PA, USA.[15]Emory University, Atlanta, GA, USA.[16]University of Maryland, Baltimore, MD, USA.[17]University of Washington, Seattle, WA, USA.[18]University of Mississippi, Jackson, MS, USA.[19]National Institutes of Health, Bethesda, MD, USA.[20]Johns Hopkins University, Baltimore, MD, USA.[21]University of Kentucky, Lexington, KY, USA.[22]Duke University, Durham, NC, USA.[23]University of Alabama, Birmingham, AL, USA.[24]Stanford University, Stanford, CA, USA.[25]Medical College of Wisconsin, Milwaukee, WI, USA.[26]Providence Health Care, Medicine, Vancouver, #N/A, Canada.[27]Baylor College of Medicine Human Genome Sequencing Center, Houston, TX, USA.[28]Cleveland Clinic, Cleveland, OH, USA.[29]Tempus, University of Colorado Anschutz Medical Campus, Aurora, CO, USA.[30]Columbia University, New York, NY, USA.[31]The Emmes Corporation, LTRC, Rockville, MD, USA.[32]Cleveland Clinic, Quantitative Health Sciences, Cleveland, OH, USA.[33]Johns Hopkins University, Medicine, Baltimore, MD, USA.[34]National Heart, Lung, and Blood Institute, National Institutes of Health, Bethesda, MD, USA.[35]Boston University, Massachusetts General Hospital, Boston University School of Medicine, Boston, MA, USA.[36]University of Pittsburgh, Pittsburgh, PA, USA.[37]Fundação de Hematologia e

Hemoterapia de Pernambuco - Hemope, Recife, Brazil.[38]University of Washington, Cardiovascular Health Research Unit, Department of Medicine, Seattle, WA, USA.[39]University of Texas Rio Grande Valley School of Medicine, Human Genetics, Brownsville, TX, USA.[40]University of Texas Health at Houston, Houston, TX, USA.[41]Wake Forest Baptist Health, Department of Biochemistry, Winston-Salem, NC, USA.[42]National Jewish Health, National Jewish Health, Denver, CO, USA.[43]Medical College of Wisconsin, Pediatrics, Milwaukee, WI, USA.[44]University of Texas Health at Houston, Pediatrics, Houston, TX, USA.[45]University of California, San Francisco, San Francisco, CA, USA.[46]Stanford University, Biomedical Data Science, Stanford, CA, USA.[47]University of Washington, Biostatistics, Seattle, WA, USA.[48]Brigham & Women's Hospital, Brigham and Women's Hospital, Boston, MA, USA.[49]University of Colorado at Denver, Denver, CO, USA.[50]Brigham & Women's Hospital, Boston, MA, USA.[51]University of Montreal, Montreal, Quebe, Canada.[52]University of Mississippi, Medicine, Jackson, MS, USA.[53]Washington State University, Pullman, WA, USA.[54]University of California, Los Angeles, Los Angeles, CA, USA.[55]Brigham & Women's Hospital, Medicine, Boston, MA, USA.[56]National Taiwan University, Taipei, Taiwan.[57]Brigham & Women's Hospital, Division of Preventive Medicine, Boston, MA, USA.[58]University of Virginia, Charlottesville, VA, USA.[59]Lundquist Institute, Torrance, CA, USA.[60]National Taiwan University, National Taiwan University Hospital, Taipei, Taiwan.[61]Cleveland Clinic, Cleveland Clinic, Cleveland, OH, USA.[62]National Health Research Institute Taiwan, Miaoli County, Taiwan.[63]Broad Institute, Metabolomics Platform, Cambridge, MA, USA.[64]Cleveland Clinic, Immunity and Immunology, Cleveland, OH, USA.[65]University of Vermont, Burlington, VT, USA.[66]University of Mississippi, Population Health Science, Jackson, MS, USA.[67]National Jewish Health, Denver, CO, USA.[68]Boston University, Biostatistics, Boston, MA, USA.[69]University of Texas Rio Grande Valley School of Medicine, Brownsville, TX, USA.[70]University of Michigan, Internal Medicine, Ann Arbor, MI, USA.[71]Vitalant Research Institute, San Francisco, CA, USA.[72]University of Illinois at Chicago, Chicago, IL, USA.[73]University of Chicago, Chicago, IL, USA.[74]Mayo Clinic, Health Quantitative Sciences Research, Rochester, MN, USA.[75]Washington University in St Louis, Department of Medicine, Cardiovascular Division, St. Louis, MO, USA.[76]University of Texas Health at Houston, Human Genetics Center, Department of Epidemiology, Human Genetics, and Environmental Sciences, Houston, TX, USA.[77]Vanderbilt University, Nashville, TN, USA.[78]University of Cincinnati, Cincinnati, OH, USA.[79]University of North Carolina, Chapel Hill, NC, USA.[80]University of Texas Rio Grande Valley School of Medicine, Edinburg, TX, USA.[81]Washington University in St Louis, Genetics, St Louis, MO, USA.[82]Brown University, Providence, RI, USA.[83]Harvard University, Channing Division of Network Medicine, Cambridge, MA, USA.[84]Massachusetts General Hospital, Boston, MA, USA.[85]National Jewish Health, Center for Genes, Environment and Health, Denver, CO, USA.[86]University of North Carolina, Epidemiology, Chapel Hill, NC, USA.[87]Washington University in St Louis, St Louis, MO, USA.[88]Fred Hutchinson Cancer Research Center, Seattle, WA, USA.[89]New York Genome Center, New York City, NY, USA.[90]Icahn School of Medicine at Mount Sinai, New York, NY, USA.[91]Beth Israel Deaconess Medical Center, Boston, MA, USA.[92]Boston Children's Hospital, Harvard Medical School, Department of Psychiatry, Boston, MA, USA.[93]University of Texas Rio Grande Valley School of Medicine, San Antonio, TX, USA.[94]University of Colorado Anschutz Medical Campus, Aurora, CO, USA.[95]Mass General Brigham, Obstetrics and Gynecology, Boston, MA, USA.[96]Indiana University, OB/GYN, Indianapolis, IN, USA.[97]University of Mississippi, Cardiology, Jackson, MS, USA.[98]University of Calgary, Medicine, Calgary, Alberta, Canada.[99]University of Maryland, Genetics, Philadelphia, PA, USA.[100]Yale University, Department of Chronic Disease Epidemiology, New Haven, CT, USA.[101]Tulane University, New Orleans, LA, USA.[102]University of Washington, Epidemiology, Seattle, WA, USA.[103]Wake Forest Baptist Health, Winston-Salem, NC, USA.[104]Brigham & Women's Hospital, Channing Division of Network Medicine,

Boston, MA, USA.[105]University of Iowa, Iowa City, IA, USA.[106]National Health Research Institute Taiwan, Institute of Population Health Sciences, NHRI, Miaoli County, Taiwan.[107]Tri-Service General Hospital National Defense Medical Center, Taiwan.[108]Blood Works Northwest, Seattle, WA, USA.[109]Taichung Veterans General Hospital Taiwan, Taichung City, Taiwan.[110]Oklahoma State University Medical Center, Internal Medicine, DIvision of Endocrinology, Diabetes and Metabolism, Columbus, OH, USA.[111]National Heart, Lung, and Blood Institute, National Institutes of Health, NHLBI, Bethesda, MD, USA.[112]Blood Works Northwest, Research Institute, Seattle, WA, USA.[113]University of Michigan, Biostatistics, Ann Arbor, MI, USA.[114]Albert Einstein College of Medicine, New York, NY, USA.[115]Harvard University, Cambridge, MA, USA.[116]McGill University, Montréal, Quebec, Canada.[117]University of Colorado at Denver, Epidemiology, Aurora, CO, USA.[118]Blood Works Northwest, Medicine, Seattle, WA, USA.[119]Loyola University, Public Health Sciences, Maywood, IL, USA.[120]Harvard School of Public Health, Biostats, Boston, MA, USA.[121]University of Colorado at Denver, Medicine, Aurora, CO, USA.[122]Boston University, University of Massachusetts Chan Medical School, Worcester, MA, USA.[123]Harvard School of Public Health, Boston, MA, USA.[124]Brown University, Epidemiology and Medicine, Providence, RI, USA.[125]Duke University, Cardiology, Durham, NC, USA.[126]Stanford University, Cardiovascular Institute, Stanford, CA, USA.[127]Icahn School of Medicine at Mount Sinai, The Charles Bronfman Institute for Personalized Medicine, New York, NY, USA.[128]Boston University, Boston, MA, USA.[129]Ohio State University, Division of Pulmonary, Critical Care and Sleep Medicine, Columbus, OH, USA.[130]Broad Institute, Harvard University, Massachusetts General Hospital, Boston, MA, USA.[131]George Washington University, cardiology, Washington DC, USA.[132]University of Alabama, University of Alabama at Birmingham, Birmingham, AL, USA.[133]Brown University, Epidemiology, Providence, RI, USA.[134]University of Washington, Genome Sciences, Seattle, WA, USA.[135]RTI International, Research Triangle Park, Durham, NC, USA.[136]Massachusetts General Hospital, Medicine, Boston, MA, USA.[137]University of Arizona, Tucson, AZ, USA.[138]Stanford University, Center For Sleep Sciences and Medicine, Palo Alto, CA, USA.[139]National Institute of Child Health and Human Development, National Institutes of Health, Bethesda, MD, USA.[140]Oklahoma Medical Research Foundation, Genes and Human Disease, Oklahoma City, OK, USA.[141]Ministry of Health, Government of Samoa, Apia, Samoa.[142]Howard University, Washington DC, USA.[143]University of Washington, Department of Genome Sciences, Seattle, WA, USA.[144]University of Maryland, Balitmore, MD, USA.[145]University at Buffalo, Buffalo, NY, USA.[146]University of Pennsylvania, Division of Sleep Medicine/Department of Medicine, Philadelphia, PA, USA.[147]Stanford University, Stanford Cardiovascular Institute, Stanford, CA, USA.[148]Wake Forest Baptist Health, Biochemistry, Winston-Salem, NC, USA.[149]University of Minnesota, Minneapolis, MN, USA.[150]RTI International, Biostatistics and Epidemiology Division, Research Triangle Park, NC, USA.[151]Boston University, Department of Biostatistics, Boston, MA, USA.[152]Fred Hutchinson Cancer Research Center, Fred Hutch and UW, Seattle, WA, USA.[153]Johns Hopkins University, Cardiology/Medicine, Baltimore, MD, USA.[154]University of Colorado at Denver, Medicine, Denver, CO, USA.[155]University of Colorado at Denver, CCPM, Denver, CO, USA.[156]University of North Carolina, Genetics, Chapel Hill, NC, USA.[157]Northwestern University, Chicago, IL, USA.[158]New York Genome Center, New York Genome Center, New York City, NY, USA.[159]Fred Hutchinson Cancer Research Center, University of Washington, Seattle, WA, USA.[160]Lutia I Puava Ae Mapu I Fagalele, Apia, Samoa.[161]University of Ottawa, Sleep Research Unit, University of Ottawa Institute for Mental Health Research, Ottawa, Ontario, Canada.[162]Vanderbilt University, Medicine, Pharmacology, Biomedicla Informatics, Nashville, TN, USA.[163]Lundquist Institute, Pediatrics, Torrance, CA, USA.[164]Universidade de Sao Paulo, Faculdade de Medicina, Sao Paulo, Brazil.[165]University of Maryland, Pathology, Seattle, WA, USA.[166]Lundquist Institute, TGPS, Torrance, CA, USA.[167]Harvard University,

Division of Hematology/Oncology, Boston, MA, USA.[168]Harvard Medical School, Genetics, Boston, MA, USA.[169]Harvard Medical School, Boston, MA, USA.[170]Université Laval, Quebec City, Canada.[171]Emory University, Pediatrics, Atlanta, GA, USA.[172]Emory University, Human Genetics, Atlanta, GA, USA.[173]Vanderbilt University, Medicine/Cardiology, Nashville, TN, USA.[174]UMass Memorial Medical Center, Worcester, MA, USA.[175]University of Saskatchewan, Saskatoon, Saskatchewan, Canada.[176]Wake Forest Baptist Health, Biostatistical Sciences, Winston-Salem, NC, USA.[177]University of Colorado at Denver, Genomic Cardiology, Aurora, CO, USA.[178]Brigham & Women's Hospital, Channing Department of Medicine, Boston, MA, USA.[179]Stanford University, Genetics, Stanford, CA, USA.[180]Lundquist Institute, Institute for Translational Genomics and Populations Sciences, Torrance, CA, USA.[181]University of Washington, University of Washington, Department of Genome Sciences, Seattle, WA, USA.[182]Fred Hutchinson Cancer Research Center, Cancer Prevention Division of Public Health Sciences, Seattle, WA, USA.[183]University of Pennsylvania, Genetics, Philadelphia, PA, USA.[184]University of Alabama, Biostatistics, Birmingham, AL, USA.[185]University of Washington, Department of Biostatistics, Seattle, WA, USA.[186]University of Vermont, Pathology & Laboratory Medicine, Burlington, VT, USA.[187]University of Southern California, USC Methylation Characterization Center, University of Southern California, CA, USA.[188]Brigham & Women's Hospital, Mass General Brigham, Boston, MA, USA.[189]Brigham & Women's Hospital, Channing Division of Network Medicine, Department of Medicine, Boston, MA, USA.[190]Indiana University, Epidemiology, Indianapolis, IN, USA.[191]Henry Ford Health System, Detroit, MI, USA.[192]Beth Israel Deaconess Medical Center, Cardiology, Cambridge, MA, USA.[193]University of Pittsburgh, Medicine, Pittsburgh, PA, USA.[194]University of Michigan, Department of Epidemiology, Ann Arbor, MI, USA.[195]Case Western Reserve University, Department of Population and Quantitative Health Sciences, Cleveland, OH, USA.
