# Supplementary material for: GAWMerge expands GWAS sample size and diversity by combining array-based genotyping and whole-genome sequencing
Source: Commun Biol. 2022 Aug 11;5:806. doi: 10.1038/s42003-022-03738-6 (PMC9372058; doi:10.1038/s42003-022-03738-6)
Supplement: Supplementary file 3 — Reporting Summary [file 42003_2022_3738_MOESM3_ESM.pdf]

Corresponding author(s): Eric Otto Johnson
Last updated by author(s): May 26, 2022

nature portfolio

Reporting Summary

Nature Portfolio wishes to improve the reproducibility of the work that we publish. This form provides structure for consistency and transparency in reporting. For further information on Nature Portfolio policies, see our Editorial Policies and the Editorial Policy Checklist.

Statistics

For all statistical analyses, confirm that the following items are present in the figure legend, table legend, main text, or Methods section.

n/a	Confirmed	
☐	☒	The exact sample size (*n*) for each experimental group/condition, given as a discrete number and unit of measurement
☐	☒	A statement on whether measurements were taken from distinct samples or whether the same sample was measured repeatedly
☐	☒	The statistical test(s) used AND whether they are one- or two-sided<br>*Only common tests should be described solely by name; describe more complex techniques in the Methods section.*
☐	☒	A description of all covariates tested
☐	☒	A description of any assumptions or corrections, such as tests of normality and adjustment for multiple comparisons
☐	☒	A full description of the statistical parameters including central tendency (e.g. means) or other basic estimates (e.g. regression coefficient) AND variation (e.g. standard deviation) or associated estimates of uncertainty (e.g. confidence intervals)
☐	☒	For null hypothesis testing, the test statistic (e.g. *F*, *t*, *r*) with confidence intervals, effect sizes, degrees of freedom and *P* value noted<br>*Give P values as exact values whenever suitable.*
☒	☐	For Bayesian analysis, information on the choice of priors and Markov chain Monte Carlo settings
☐	☒	For hierarchical and complex designs, identification of the appropriate level for tests and full reporting of outcomes
☐	☒	Estimates of effect sizes (e.g. Cohen's *d*, Pearson's *r*), indicating how they were calculated

Our web collection on statistics for biologists contains articles on many of the points above.

Software and code

Policy information about availability of computer code

Data collection	No primary data collection was done for this study
Data analysis	All software used in this study is publicly available, including:<br>- Plink: https://zzz.bwh.harvard.edu/plink/<br>- BCFTools: https://samtools.github.io/bcftools/bcftools.html<br>- ShapeIt2: https://mathgen.stats.ox.ac.uk/genetics_software/shapeit/shapeit.html<br>- Minimac4: https://genome.sph.umich.edu/wiki/Minimac4<br>- rvTests: http://zhanxw.github.io/rvtests/

For manuscripts utilizing custom algorithms or software that are central to the research but not yet described in published literature, software must be made available to editors and reviewers. We strongly encourage code deposition in a community repository (e.g. GitHub). See the Nature Portfolio guidelines for submitting code & software for further information.

Data

Policy information about availability of data

All manuscripts must include a data availability statement. This statement should provide the following information, where applicable:

- Accession codes, unique identifiers, or web links for publicly available datasets
- A description of any restrictions on data availability
- For clinical datasets or third party data, please ensure that the statement adheres to our policy

The individual-level genotype and phenotype data used are all available through dbGaP. The dbGap study accession number for COGEND is phs000404, for

COPDGene are phs000179 (parent study with array genotype data) and phs000951 (WGS data generated by TOPMed), and for ECLIPSE are phs001252 (parent study with array genotype data) and phs001472 (WGS data generated by TOPMed).

Field-specific reporting

Please select the one below that is the best fit for your research. If you are not sure, read the appropriate sections before making your selection.

☒ Life sciences ☐ Behavioural & social sciences ☐ Ecological, evolutionary & environmental sciences

For a reference copy of the document with all sections, see nature.com/documents/nr-reporting-summary-flat.pdf

Life sciences study design

All studies must disclose on these points even when the disclosure is negative.

Sample size	Samples sizes were determined based on the availability of data and how to best show unbias results for our protocol for integrating WGS and array genotyping data. For example to asses control of type-I error with our integration protocol, we used smoking-related datasets and split the COPDGene cohort as described in the Results section therefore not receiving a smoking-related signals in our testing.
Data exclusions	In our evaluation of the GAWMerge protocol for integrating WGS and array genotyping data, we describe data exclusion criteria in the Results and Methods section of our manuscript.
Replication	In our evaluation of the GAWMerge protocol for integrating WGS and array genotyping data, we replicate known COPD GWAS hits comparing our results to the published work by the COPDGene cohort.
Randomization	To maximize the COPDGene cohort in the evaluation of type-I error of our GAWMerge protocol, we randomized the samples into two subsets as described in the Results section of the manuscript. In our other evaluations randomization was not necessary.
Blinding	Blinding is not relevant to this manuscript work as it is a method for integrating WGS and array genotyping data for genome-wide association study.

Reporting for specific materials, systems and methods

We require information from authors about some types of materials, experimental systems and methods used in many studies. Here, indicate whether each material, system or method listed is relevant to your study. If you are not sure if a list item applies to your research, read the appropriate section before selecting a response.

Materials & experimental systems

n/a	Involved in the study	
☒	☐	Antibodies
☒	☐	Eukaryotic cell lines
☒	☐	Palaeontology and archaeology
☒	☐	Animals and other organisms
☐	☒	Human research participants
☒	☐	Clinical data
☒	☐	Dual use research of concern

Methods

n/a	Involved in the study	
☒	☐	ChIP-seq
☒	☐	Flow cytometry
☒	☐	MRI-based neuroimaging

Human research participants

Policy information about studies involving human research participants

Population characteristics	Genome-Wide association analysis was conducted with ten principal components as covariates.
Recruitment	No recruitment was conducted for obtaining the data used in the manuscript. Descriptions of recruitment strategies that generated the available data as described within the data availability statement and dbGaP.
Ethics oversight	The use of the WGS TOPMed data was approved by the TOPMed Methods working group. Data approval of the dbGaP available data was approved by the RTI International Institutional Review Board.

Note that full information on the approval of the study protocol must also be provided in the manuscript.
